# Supplementary figures and images for: Synaptobrevin2 monomers and dimers differentially engage to regulate the functional trans-SNARE assembly
Source: Life Sci Alliance. 2024 Jan 18;7(4):e202402568. doi: 10.26508/lsa.202402568 (PMC10796598; doi:10.26508/lsa.202402568)

SourceDataF1

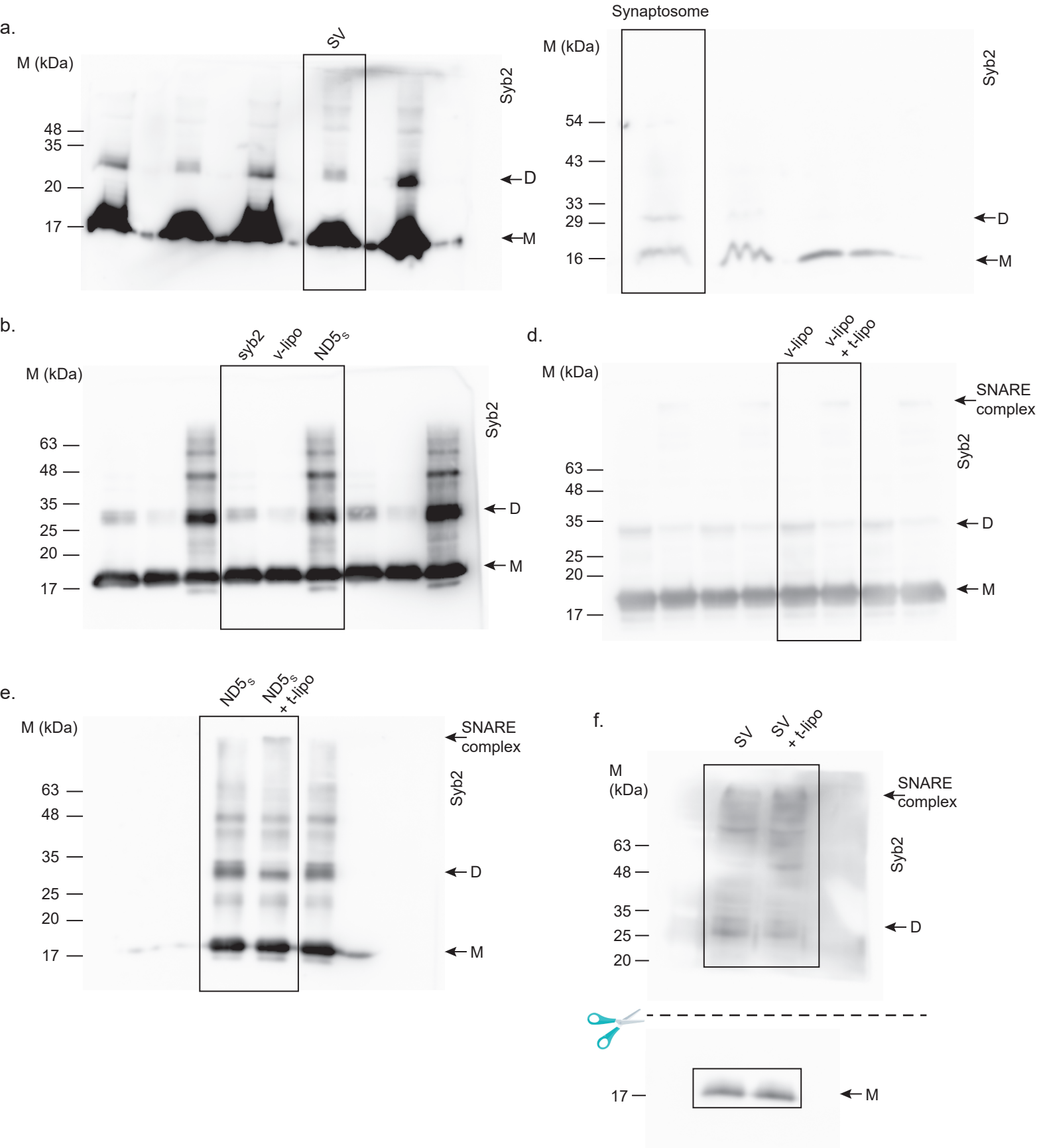

Supplement: Supplementary file 1 [file LSA-2024-02568_SdataF1.pdf]

# SourceDataFS1

a.

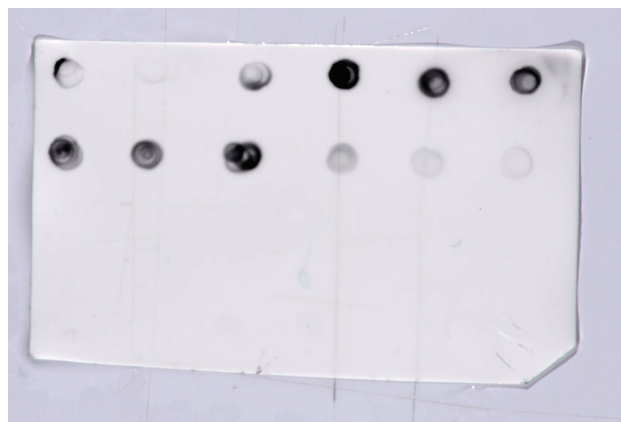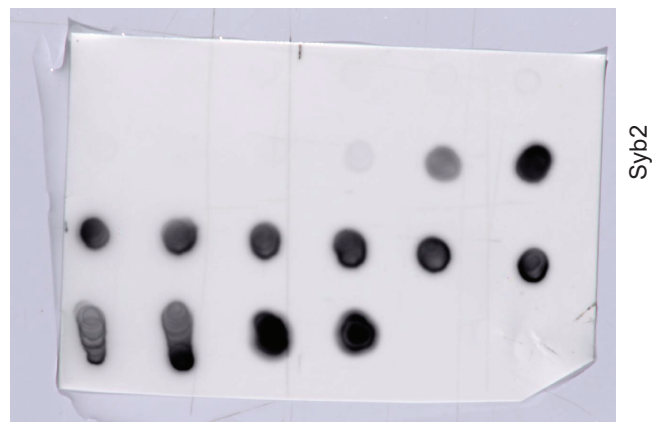

b.

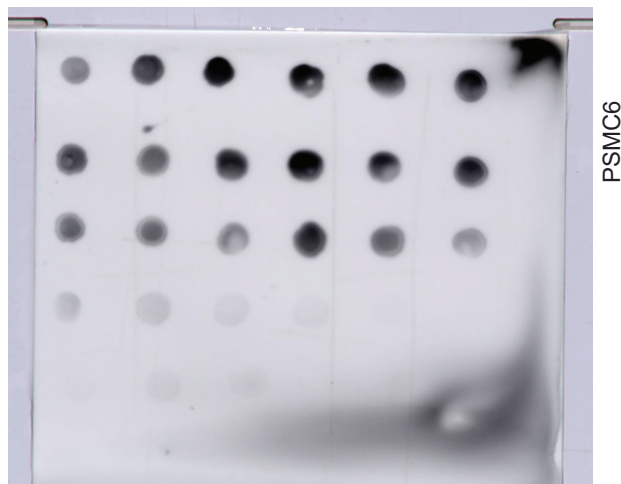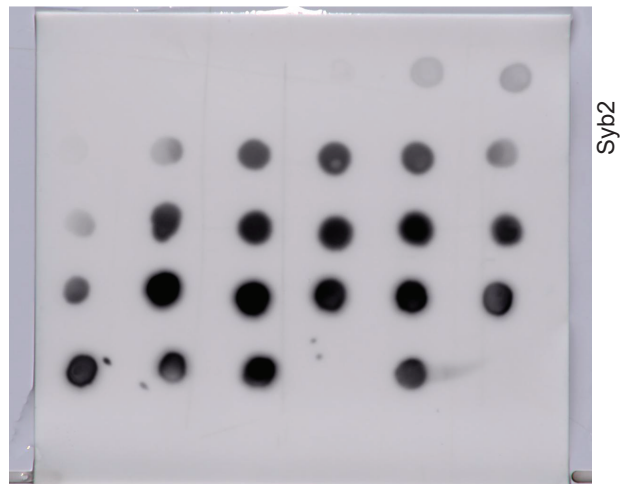

c.

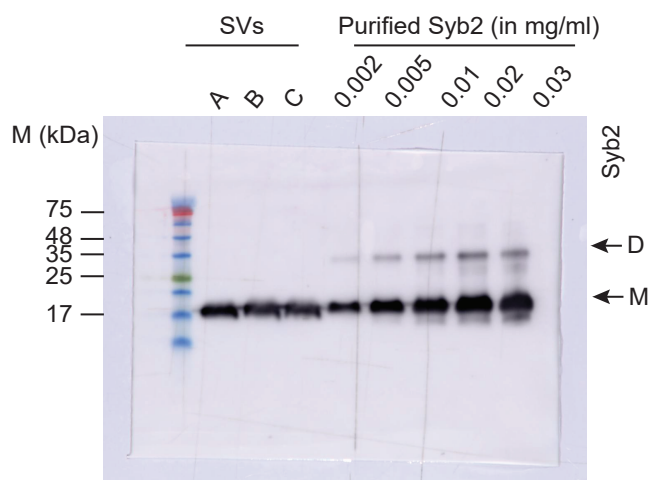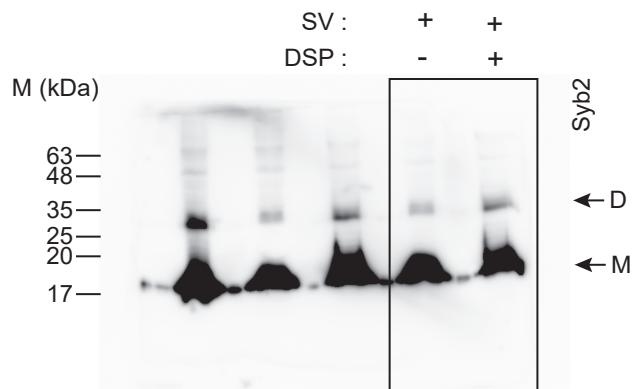

Supplement: Supplementary file 2 [file LSA-2024-02568_SdataFS1.pdf]

SourceDataFS2

b.

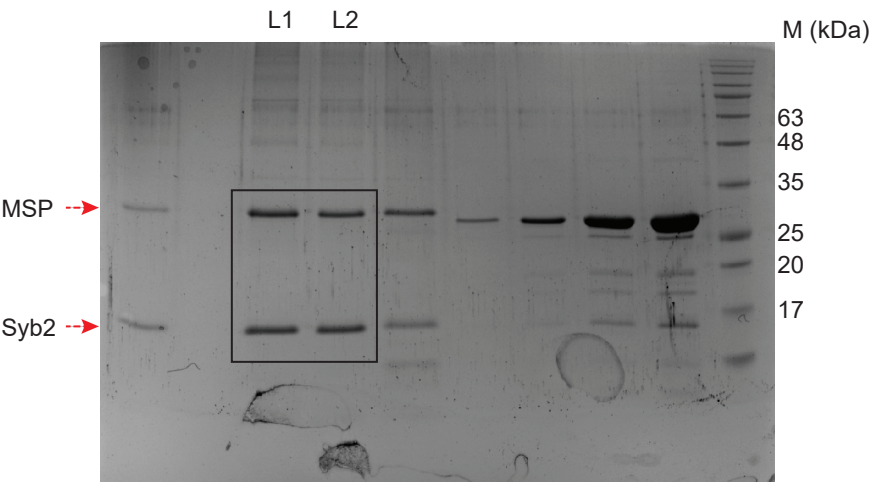

Supplement: Supplementary file 3 [file LSA-2024-02568_SdataFS2.pdf]

SourceDataFS4

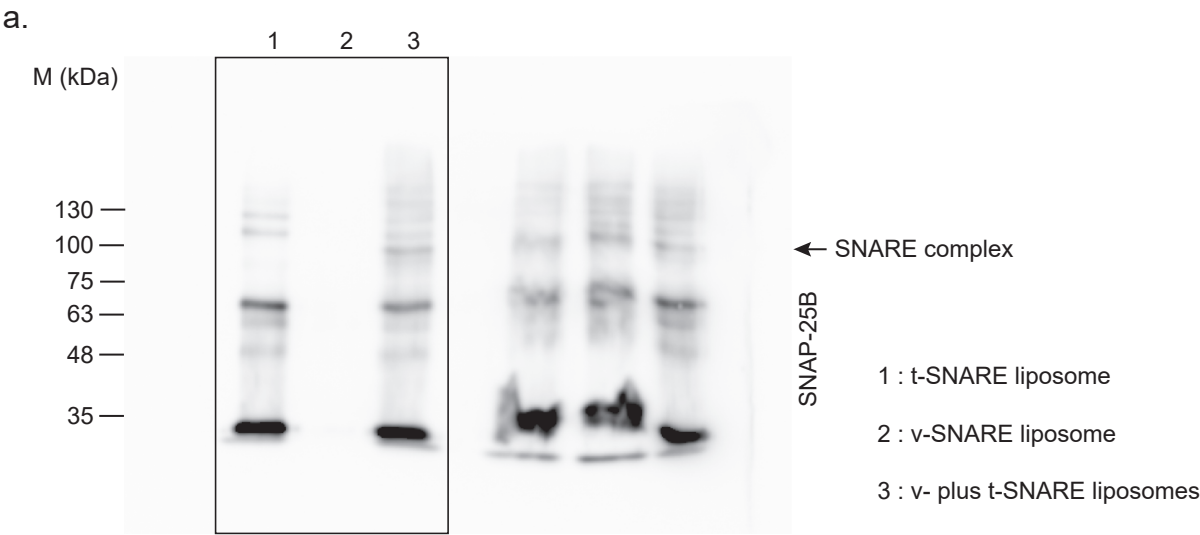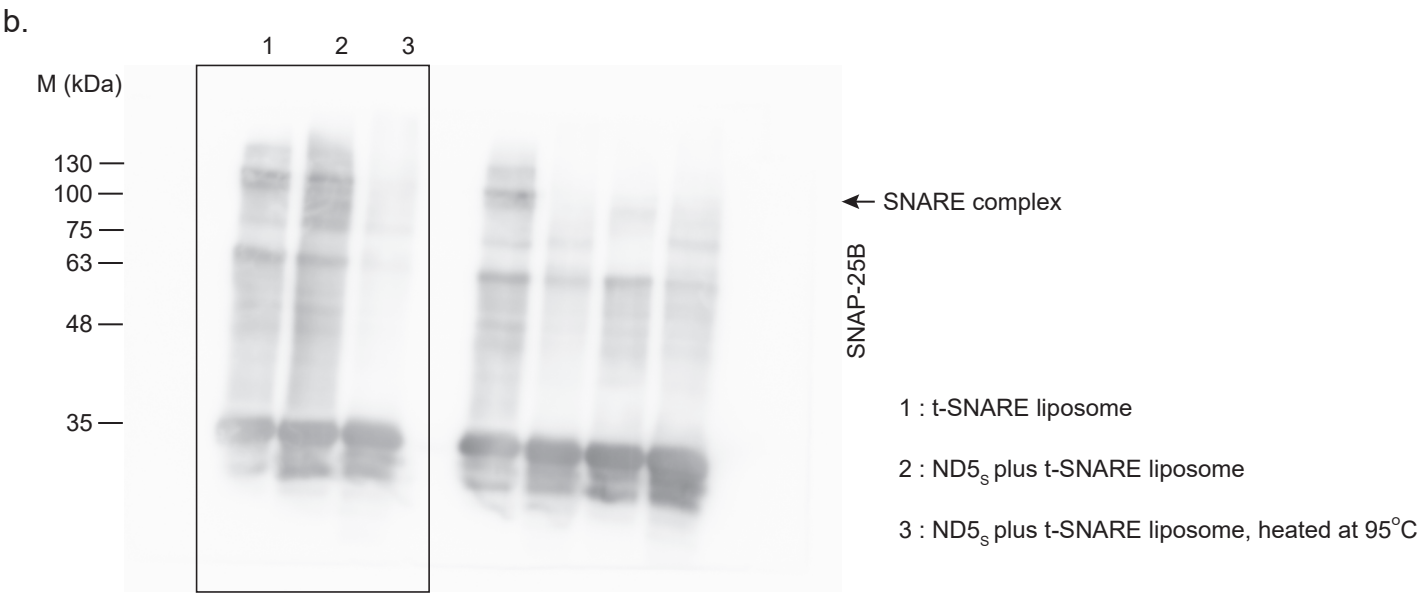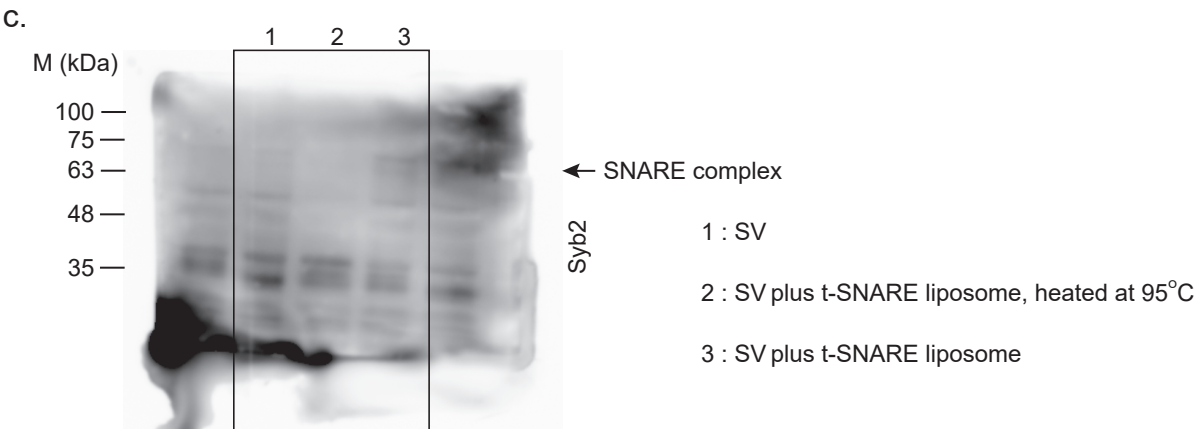

Supplement: Supplementary file 4 [file LSA-2024-02568_SdataFS4.pdf]

SourceDataF2

d.

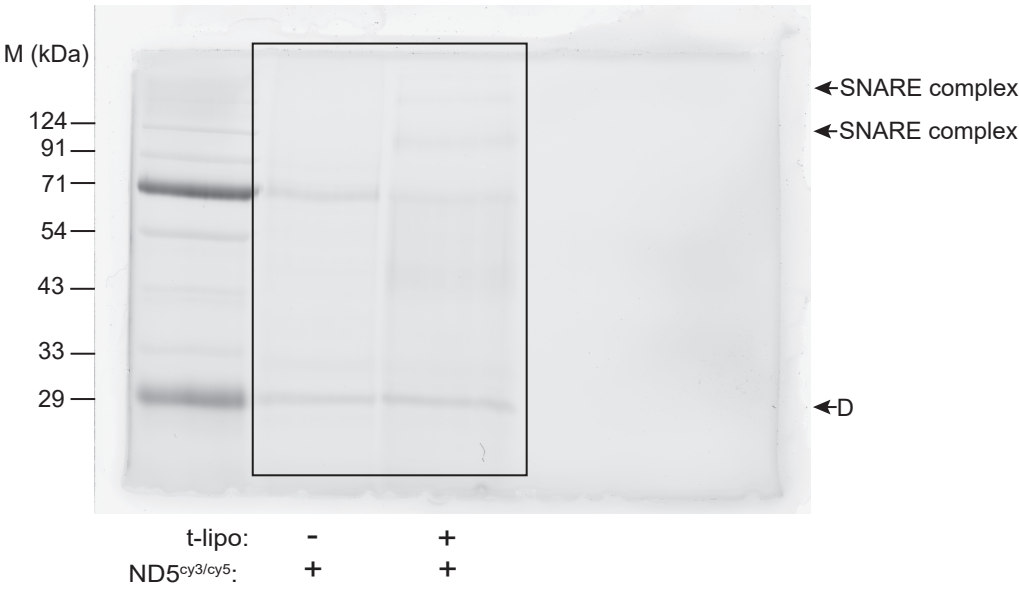

Supplement: Supplementary file 5 [file LSA-2024-02568_SdataF2.pdf]

SourceDataF3

a.

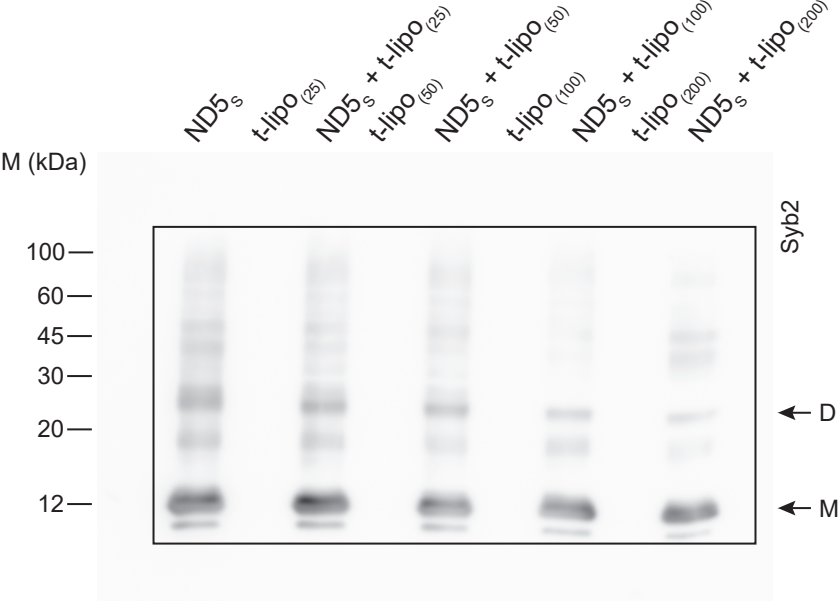

c.

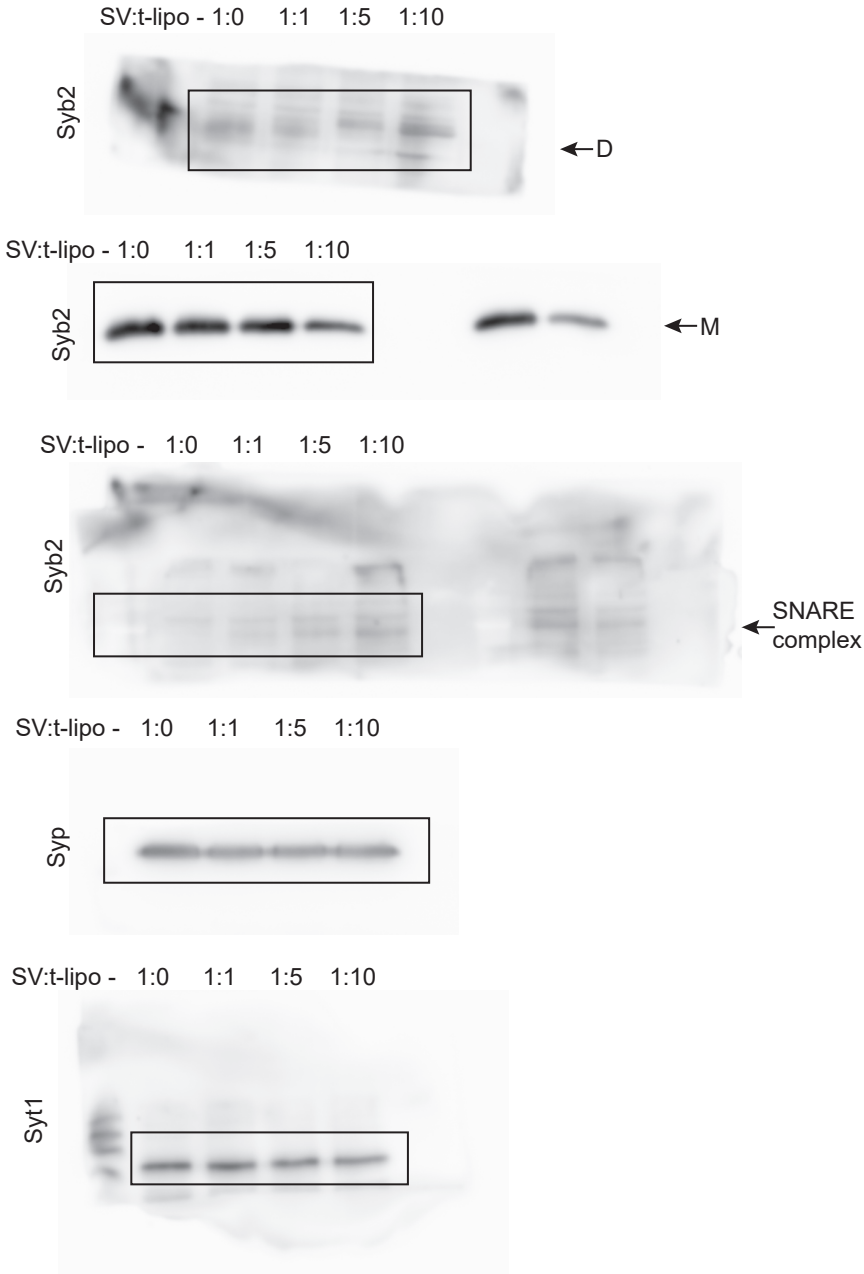

Supplement: Supplementary file 6 [file LSA-2024-02568_SdataF3.pdf]

SourceDataF4

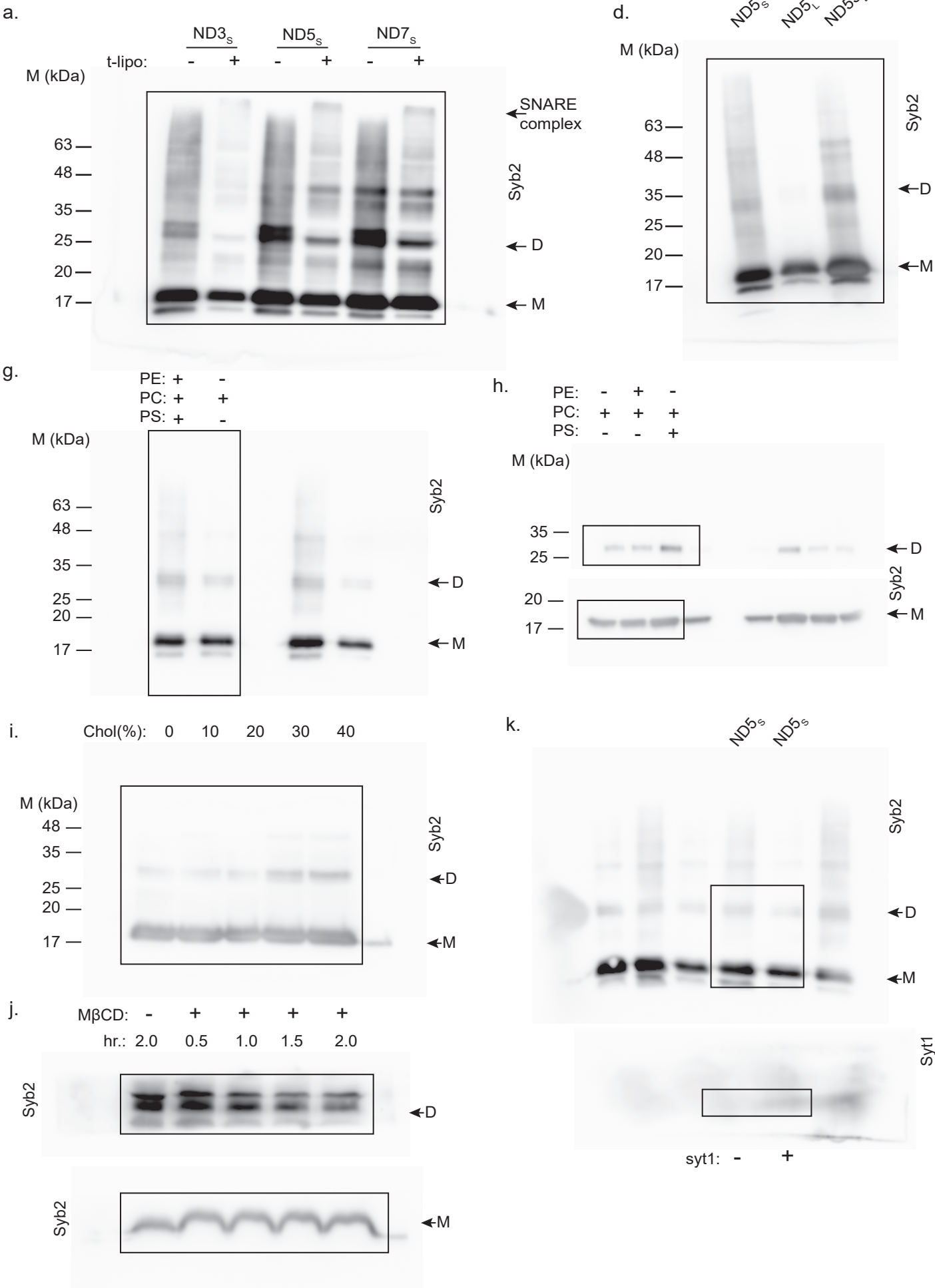

Supplement: Supplementary file 7 [file LSA-2024-02568_SdataF4.pdf]

SourceDataFS8

a.

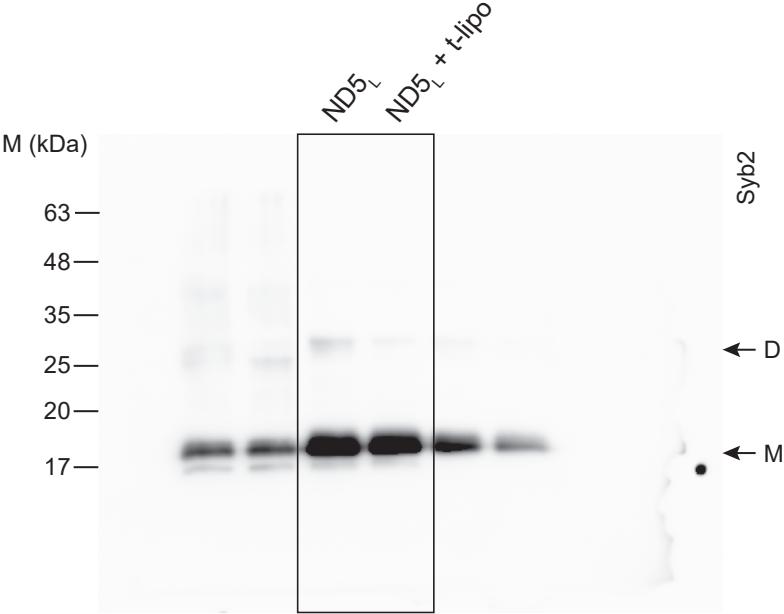

b.

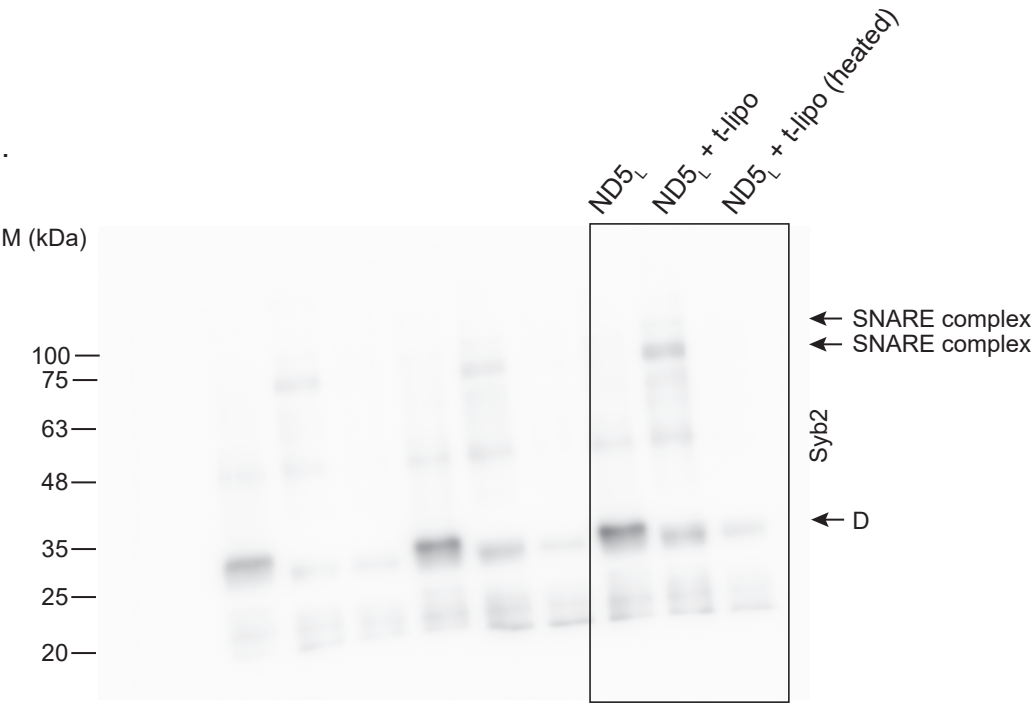

Supplement: Supplementary file 8 [file LSA-2024-02568_SdataFS8.pdf]

SourceDataF6

b.

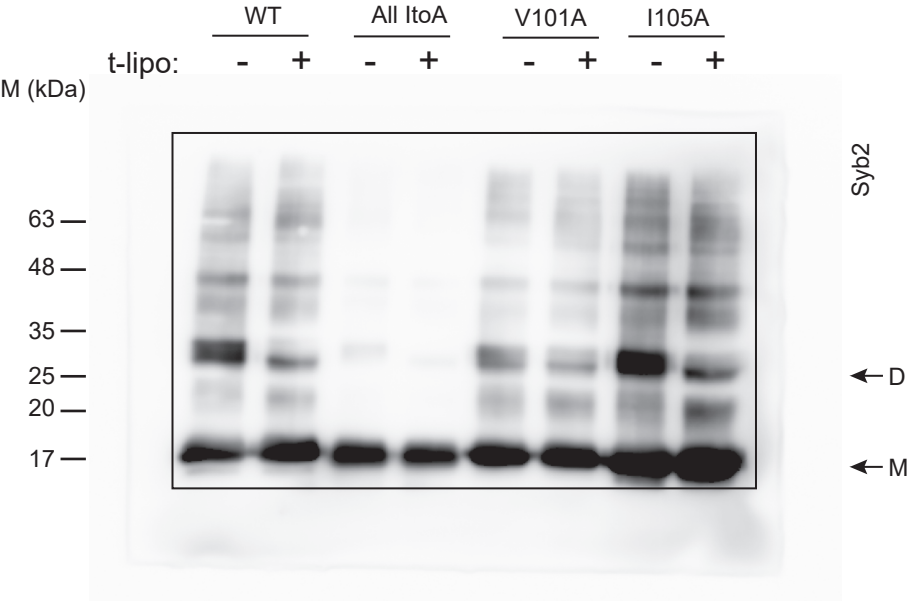

Supplement: Supplementary file 9 [file LSA-2024-02568_SdataF6.pdf]

SourceDataFS10

a.

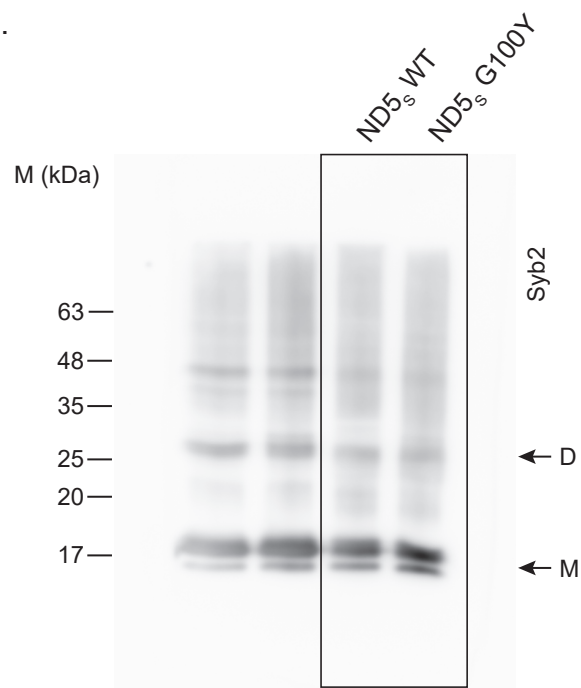

b.

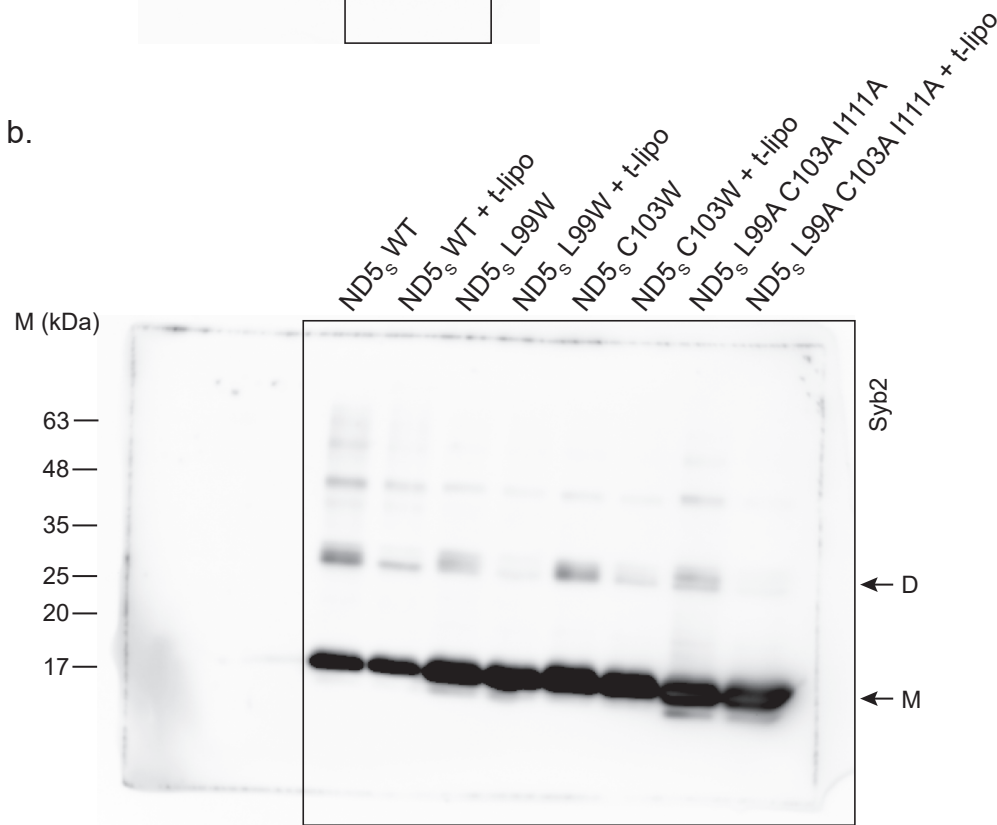

Supplement: Supplementary file 10 [file LSA-2024-02568_SdataFS10.pdf]

SourceDataFS11

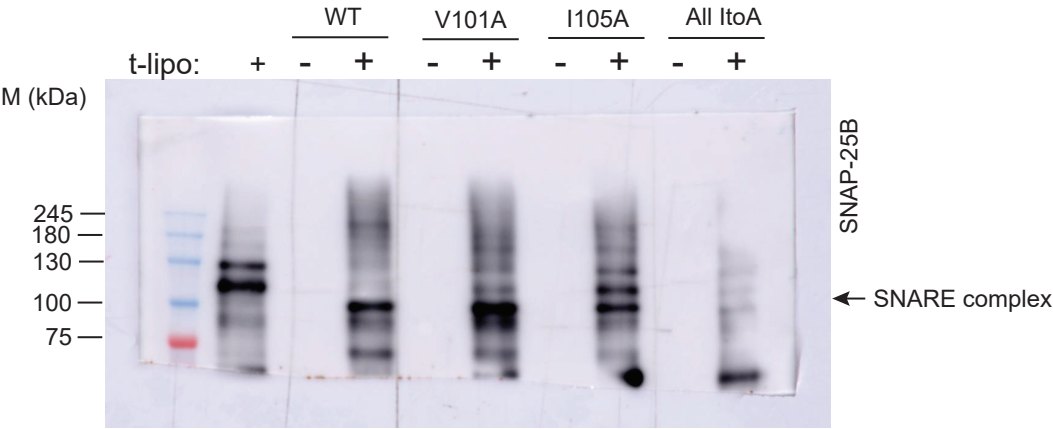

Supplement: Supplementary file 11 [file LSA-2024-02568_SdataFS11.pdf]
