# Supplementary material for: Synaptobrevin2 monomers and dimers differentially engage to regulate the functional trans-SNARE assembly
Source: Life Sci Alliance. 2024 Jan 18;7(4):e202402568. doi: 10.26508/lsa.202402568 (PMC10796598; doi:10.26508/lsa.202402568)
Supplement: Supplementary file 12 [file LSA-2024-02568_TableS1.docx]

| Model | ExpDec2 |
| --- | --- |
| Equation | y = A1*exp(-x/t1) + A2*exp(-x/t2) + y0 |
| y0 | 0.79984 ± 0.00234 |
| A1 | 1.05812 ± 0.38867 |
| t1 | 20.21919 ± 3.63683 |
| A2 | 0.30814 ± 0.03453 |
| t2 | 88.72358 ± 7.3607 |
| R-Square (COD) | 0.99563 |

**Table S1**
