# Supplementary material for: Synaptobrevin2 monomers and dimers differentially engage to regulate the functional trans-SNARE assembly
Source: Life Sci Alliance. 2024 Jan 18;7(4):e202402568. doi: 10.26508/lsa.202402568 (PMC10796598; doi:10.26508/lsa.202402568)
Supplement: Supplementary file 13 [file LSA-2024-02568_TableS2.docx]

| Antibody (Ab) | Catalogue No | Company | Dilution |
| --- | --- | --- | --- |
| SNAP-25 Rabbit mAb | ab109105 | Abcam (UK) | 1:3000 |
| Syb2 Rabbit mAb | 13508S | Cell Signaling Technology (Danvers, MA) | 1:3000 |
| PSMC6 Mouse mAb | ab22639 | Abcam (UK) | 1:3000 |
| Syp Rabbit mAb | 36406S | Cell Signaling Technology (Danvers, MA) | 1:3000 |
| Syt1 Mouse mAb | ab13259 | Abcam (UK) | 1:3000 |
| Hsc70 Rabbit mAb | ab51052 | Abcam (UK) | 1:3000 |
| Anti-rabbit IgG, HRP linked | 7074S | Cell Signaling Technology (Danvers, MA) | 1:3000 |
| Anti-mouse IgG, HRP linked | 7076S | Cell Signaling Technology (Danvers, MA) | 1:3000 |

**Table S2**
